# Pronounced Effects of HERG-Blockers E-4031 and Erythromycin on APD, Spatial APD Dispersion and Triangulation in Transgenic Long-QT Type 1 Rabbits

**DOI:** 10.1371/journal.pone.0107210

**Published:** 2014-09-22

**Authors:** David Ziupa, Julia Beck, Gerlind Franke, Stefanie Perez Feliz, Maximilian Hartmann, Gideon Koren, Manfred Zehender, Christoph Bode, Michael Brunner, Katja E. Odening

**Affiliations:** 1 Heart Center University of Freiburg, Department of Cardiology and Angiology I, Freiburg, Germany; 2 Cardiovascular Research Center, Division of Cardiology, Rhode Island Hospital, Alpert Medical School of Brown University, Providence, Rhode Island, United States of America; University of Bologna & Italian Institute of Technology, Italy

## Abstract

**Background:**

Prolongation of action potential duration (APD), increased spatial APD dispersion, and triangulation are major factors promoting drug-induced ventricular arrhythmia. Preclinical identification of HERG/*I_Kr_*-blocking drugs and their pro-arrhythmic potential, however, remains a challenge. We hypothesize that transgenic long-QT type 1 (LQT1) rabbits lacking repolarizing *I_Ks_* current may help to sensitively detect HERG/*I_Kr_*-blocking properties of drugs.

**Methods:**

Hearts of adult female transgenic LQT1 and wild type littermate control (LMC) rabbits were Langendorff-perfused with increasing concentrations of HERG/*I_Kr_*-blockers E-4031 (0.001–0.1 µM, n = 9/7) or erythromycin (1–300 µM, n = 9/7) and APD, APD dispersion, and triangulation were analyzed.

**Results:**

At baseline, APD was longer in LQT1 than in LMC rabbits in LV apex and RV mid. Erythromycin and E-4031 prolonged APD in LQT1 and LMC rabbits in all positions. However, erythromycin-induced percentaged APD prolongation related to baseline (%APD) was more pronounced in LQT1 at LV base-lateral and RV mid positions (100 µM, LQT1, +40.6±9.7% vs. LMC, +24.1±10.0%, p<0.05) and E-4031-induced %APD prolongation was more pronounced in LQT1 at LV base-lateral (0.01 µM, LQT1, +29.6±10.6% vs. LMC, +19.1±3.8%, p<0.05) and LV base-septal positions. Moreover, erythromycin significantly increased spatial APD dispersion only in LQT1 and increased triangulation only in LQT1 in LV base-septal and RV mid positions. Similarly, E-4031 increased triangulation only in LQT1 in LV apex and base-septal positions.

**Conclusions:**

E-4031 and erythromycin prolonged APD and increased triangulation more pronouncedly in LQT1 than in LMC rabbits. Moreover, erythromycin increased APD dispersion only in LQT1, indicating that transgenic LQT1 rabbits could serve as sensitive model to detect HERG/*I_Kr_*-blocking properties of drugs.

## Introduction

Long-QT syndromes (LQTS) are characterized by prolonged cardiac repolarization, resulting in QT interval prolongation, the occurrence of torsade de pointes (TdP) tachycardia and an increased risk for sudden cardiac death [1]. Beside the relatively rare congenital form, LQTS frequently occurs as a drug-induced, acquired variant [2] with an estimated incidence of cardiac events in 2.5 to 10.9 per million per year [3], [4].

While (genetically determined) dysfunction of various cardiac potassium channels of phase-3-repolarization constitutes the predominant mechanism in congenital LQTS, the HERG potassium channel represents the main target in drug-induced LQTS [5], [6]. However, there seems to be some overlap between both syndromes, since subjects with silent LQT mutations showing normal QT intervals at baseline have a high propensity to develop drug-induced QT prolongation and TdP arrhythmias [7], [8]. Moreover, in up to 10–15% of patients with drug-induced QT prolongations and TdP arrhythmias single nucleotide polymorphisms (SNPs) have been discovered in LQT-related genes like *KCNQ1*, *KCNH2*, *SCN5A*, *KCNE1* and *KCNE2* [9], [10] and it has been demonstrated that some of these SNPs in LQTS-associated genes may increase the risk for drug-induced LQTS up to 9-fold [11]. We therefore hypothesized that models with LQTS-mutations causing a reduced repolarization reserve may be particularly sensitive to reveal the pro-arrhythmic risk of a certain drug.

Since several widely used drugs (e.g. terfenadine, astemizole, and cisapride) with HERG/*I_Kr_*-blocking properties had to be withdrawn from the market due to the occurrence of lethal ventricular arrhythmia [2], [12], all novel candidate drugs have to be screened for HERG/*I_Kr_*-blocking properties *in vitro* and *in vivo* at an early stage of drug development [13]. To date, cellular and computer models cannot sufficiently predict drug induced pro-arrhythmia [13] and tissue preparations and whole heart models still need further optimization [14]. *Ex vivo* monophasic action potential (MAP) measurements in Langendorff-perfused rabbit hearts [15], [16], [17], [18] have been established to derive action potential durations (APD) at different positions and to record arrhythmia susceptibility, combining the advantages of cellular and animal models. Since preclinical measurement of drug-induced prolongation of APD or QT intervals alone has not achieved a sufficient sensitivity for reliable detection of pro-arrhythmic drugs [19], [20], [21], additional parameters, such as spatial APD dispersion [22] and triangulation of MAP shape (indicating slowing of phase-3-repolarization) [19], [23], [24], were established to increase the performance of *ex vivo* assays for pro-arrhythmia. Reliable preclinical identification of HERG/*I_Kr_*-blocking properties of drugs and their potentially pro-arrhythmic risk, however, still remains a challenge [4], [14]. Thus, early detection of pro-arrhythmic drugs during the course of drug development is mandatory and requires refined preclinical methods [13].

We have generated transgenic long-QT type 1 (LQT1) rabbits with a dominant negative *loss-of-function* mutation (Y315S) of the human *KCNQ1* gene, resulting in an increased expression of defective KvLQT1 proteins, the α-subunit of *I_Ks_*-conducting potassium channels, and a consecutive loss of *I_Ks_* current [25]. Rabbits have pronounced similarities to humans in their cardiac ion current composition and shape of action potential [26]. Hence, LQT1 rabbits mimic the human LQTS phenotype [25]. The QT prolonging and pro-arrhythmic impact of several *I_Ks_*-blocking anesthetics [27] and HERG/*I_Kr_*-blocker dofetilide [28] has already been studied in LQTS rabbits *in vivo*.

We aimed at investigating whether transgenic LQT1 rabbits might be a sensitive, novel tool for the detection of HERG/*I_Kr_*-blocking properties of drugs due to their loss of *I_Ks_* and the resulting reduced repolarization reserve [29]. Therefore, in this first *proof of concept* study, the impact of the HERG/*I_Kr_*-blocking antibiotic drug erythromycin and the class III anti-arrhythmic drug E-4031 on APD, spatial APD dispersion, and triangulation were tested. We have chosen these two substances as E-4031 is a pure HERG/*I_Kr_*-blocker used in experimental electrophysiology, whereas erythromycin is a widely used antibiotic with known, albeit low, pro-arrhythmic effects.

As one single parameter is unlikely to assess the pro-arrhythmic risk, we use three different established methods: (1.) APD prolongation, (2.) intra- and inter-ventricular dispersion of repolarization, and (3.) triangulation of the MAP.

## Methods

### Legal aspects

All animal experiments were performed in compliance with the German animal protection law (TierSchG) after approval by the Institutional Animal Care and Use Committee (Tierschutzbeauftragter) of the University of Freiburg and the local authorities (Regierungspraesidium Freiburg; animal protocol number G11/107).

### Monophasic action potential measurements

Female transgenic LQT1 and wild type littermate control (LMC) *New Zealand White* rabbits were genotyped as described [25] and the phenotype of LQT1 rabbits was verified by ECG recordings. Adult LQT1 and LMC females were subjected to monophasic action potential (MAP) measurements as described previously [16], [18].

Rabbits were anesthetized with S-ketamine/xylazine (12.5/3.5 mg/kg) IM, since this combination does not affect cardiac repolarization [27]. After administration of heparin (500 IU) and thiopental-sodium (40 mg/kg) IV, hearts were rapidly excised and mounted to the Langendorff-apparatus (Model IH5, Hugo Sachs Elektronik, Hugstetten, Germany). Hearts were retrogradely perfused with warmed (37°C), oxygenated, modified Krebs-Henseleit solution (as described in [16]). A latex balloon tipped pressure transducer was introduced into the left ventricle (LV) and LV systolic and diastolic pressure and ECG were monitored continuously during the experiment.

To allow a slow ventricular pacing at 2 Hz at the base lateral LV (120 bpm; HSE Simulator C, type 224, Hugo Sachs Electronic, Harvard Apparatus GmbH, Hugstetten, Germany), the AV node was mechanically ablated. Five electrodes were positioned at the LV in apical, mid, base-lateral and base-septal position and at the right ventricle (RV mid). Epicardial MAP were recorded at baseline and during perfusion with increasing concentrations of HERG/*I_Kr_*-blockers E-4031 (0.001–0.1 µM, Sigma-Aldrich, Taufkirchen, Germany) (n = 9 LQT1, n = 7 LMC) or erythromycin (1–300 µM, Inresa Arzneimittel GmbH, Freiburg, Germany) (n = 9 LQT1, n = 7 LMC).

### Analysis and statistics

Based on RR and QT interval measured by surface ECG, heart rate corrected QT indices (QTi = QT/[86+0.22*RR]*100) were calculated, as previously described [25]. APD at 90%, 75%, and 30% of repolarization (APD_90_, APD_75_, APD_30_) were derived from the recorded MAP in all electrode positions using Isoheart Software Version 1.1.1.218 (Hugo Sachs Electronic, Harvard Apparatus GmbH, Hugstetten, Germany). Percentaged drug-induced APD_75_ prolongation related to baseline APD_75_ (%APD) was calculated to compare drug-induced APD_75_ changes between LQT1 and LMC rabbits. Spatial APD dispersion (intra-ventricular LV APD dispersion defined as standard deviation of APD in all LV segments and inter-ventricular APD dispersion defined as greatest difference between RV and LV APD), and triangulation (slowing of phase-3-repolarization; APD_90_ minus APD_30_) were calculated.

Data were only included in the analyses if good quality MAP (defined as in [18]) were measured in at least three electrode positions from baseline to highest drug concentration. Experiments of eighteen transgenic LQT1 (9 for each drug) and fourteen LMC rabbits (7 for each drug) met the inclusion criteria. There were no differences in age and weight between genotypes and among the erythromycin- or E-4031-treated groups (Table 1).

**Table 1:** Age (days) and weight (kg) of LQT1 and LMC rabbits.

Age (days)	LQT1 (n = 9)	LMC (n = 7)	Level of significance
**E4031**	172±29	153±27	*ns*
**Erythromycin**	150±30	152±33	*ns*
**Level of significance**	*ns*	*ns*	

Weight (kg)	LQT1 (n = 9)	LMC (n = 7)	Level of significance
**E4031**	3.7±0.5	3.6±0.5	*ns*
**Erythromycin**	3.6±0.6	3.3±0.4	*ns*
**Level of significance**	*ns*	*ns*	

There were no differences in age and weight among the erythromycin- or E-4031-treated groups. ns  =  not significant (p>0.1).

Statistical analyses were performed with Prism 5 (Graphpad, San Diego, USA). Significance levels of normally distributed data were analyzed using *t-test*, and the incidence of arrhythmia was analyzed with *chi-squared-test*. Levels of significance were displayed as “ns” or “not significant” for p>0.1, “t” or “trend” for p<0.1, *p<0.05, **p<0.01 and ***p<0.001.

## Results

### QT interval and action potential duration

#### Baseline

**Figure 1:**
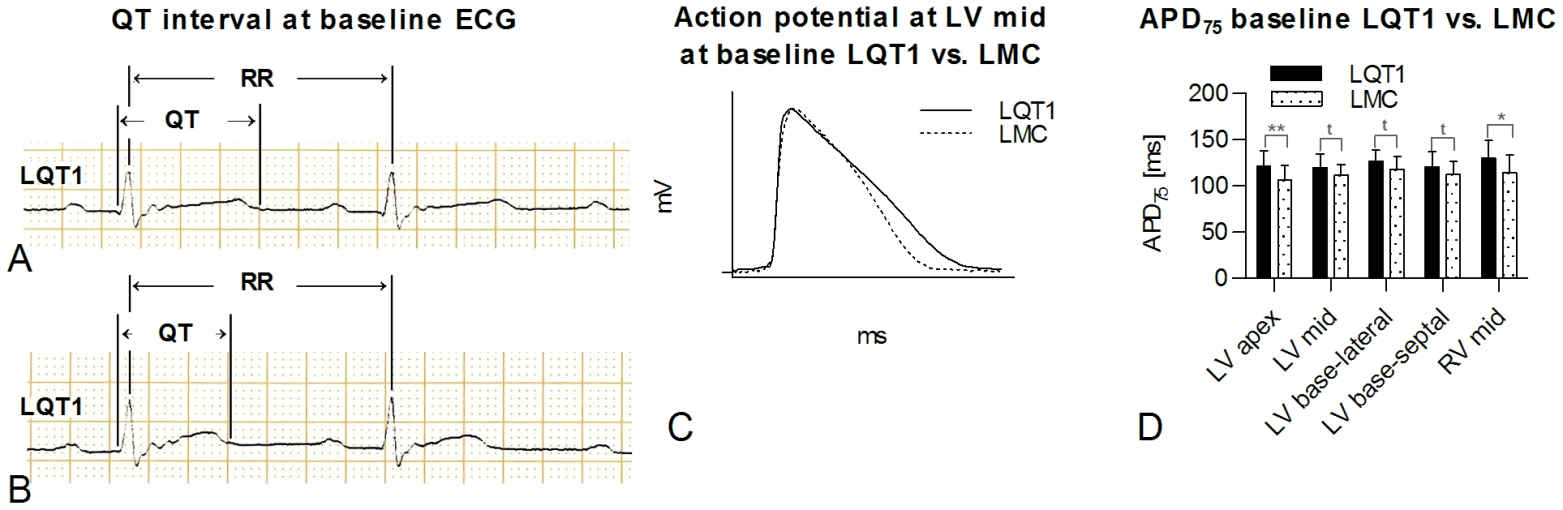
Baseline ECG and monophasic action potentials (MAP) in LQT1 and LMC rabbits. Exemplary representative baseline ECG (lead II) of (A) LQT1 and (B) LMC rabbit, QT and RR intervals are delineated. (C) Exemplary MAP of LQT1 and LMC rabbit at baseline. (D) APD in LQT1 (n = 18) vs. in LMC (n = 14) rabbits at baseline. t =  trend (p<0.1), *p<0.05, **p<0.01.

*In vivo* baseline surface ECG demonstrated significantly longer QT intervals and heart-rate corrected QT indices (QTi) in LQT1 compared to LMC rabbits (LQT1 vs. LMC; QT interval, 174.1±15.8 ms vs. 155.3±13.8 ms, p<0.05; RR interval, 348.2±37.3 ms vs. 369.4±52.8 ms, ns; QTi, 106.9±6.2% vs. 92.9±6.0%, p<0.001; Figure 1A, 1B).

Accordingly, at baseline *ex vivo* Langendorff-perfused hearts of LQT1 rabbits exhibited significantly longer APD_75_ compared to LMC in LV apex (LQT1, 121.1±16.4 ms vs. LMC, 106.0±16.1 ms, p<0.01) and RV mid positions (LQT1, 130.0±19.4 ms vs. LMC, 114.3±19.0 ms, p<0.05). In the other positions a concordant trend was noted (Figure 1C, 1D).

#### Erythromycin

**Figure 2:**
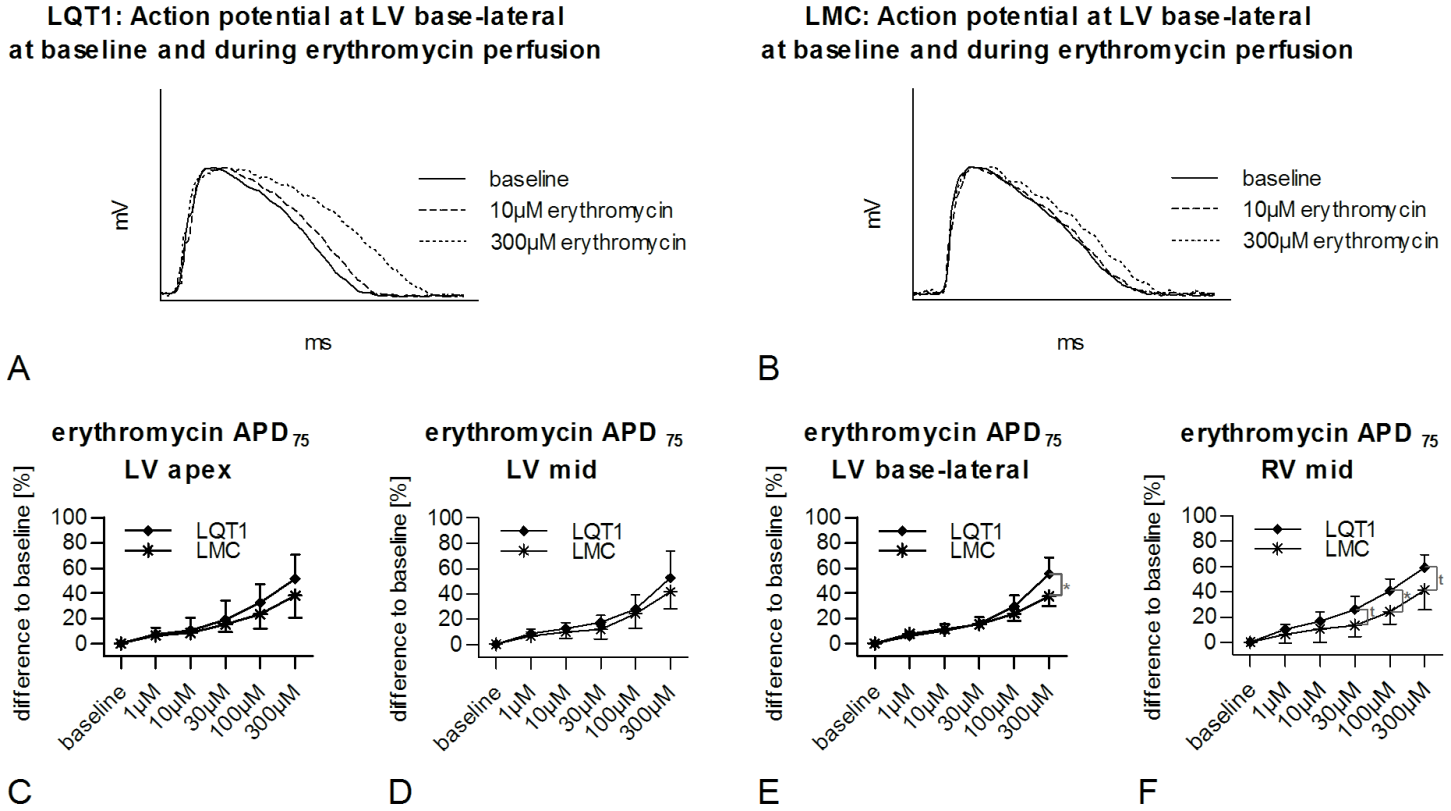
Erythromycin-induced prolongation of APD and in LQT1 and LMC rabbits. Exemplary, representative MAP in (A) LQT1 and (B) LMC rabbit at baseline and at different concentrations of erythromycin. Percentaged prolongation of APD related to baseline APD (%APD) in LQT1 (n = 9) and LMC (n = 7) rabbits in (C) LV apex, (D) LV mid, (E) LV base-lateral and (F) RV mid positions during perfusion with increasing concentrations of erythromycin. t =  trend (p<0.1), *p<0.05.

Erythromycin significantly prolonged the APD_75_ in LQT1 and LMC rabbits in all positions compared to baseline (Figure 2A, 2B). In LQT1 rabbits higher levels of significance for the increase of APD_75_ were found at low drug concentrations of erythromycin compared to LMC in all positions except for LV base-lateral (e.g. LV mid, LQT1, erythromycin vs. baseline [123.2±6.4 ms]; 10 µM, 138.4±9.1 ms, p<0.001; 300 µM, 188.0±27.9 ms, p<0.001; LMC erythromycin vs. baseline [114.5±10.4 ms]; 10 µM, 124.4±10.8 ms, p<0.01; 300 µM, 161.6±16.0 ms, p<0.001). Since baseline APD varied between LQT1 and LMC rabbits, we calculated a percentaged APD prolongation related to the baseline APD (%APD). Erythromycin-induced %APD prolongation was seen in both groups, but was more pronounced in LQT1 rabbits: in LV base-lateral position a significantly more pronounced increase of %APD was observed in LQT1 compared to LMC rabbits (300 µM, LQT1, +40.6±9.7% vs. LMC, +24.1±10.0%, p<0.05; Figure 2E). In RV mid position, this significantly greater %APD prolongation in LQT1 was already seen at lower concentration (100 µM, LQT1, +40.6±9.7% vs. LMC, +24.1±10.0%, p<0.05; Figure 2F). No pronounced differences were seen between groups in LV apex (Figure 2C), LV mid (Figure 2D) and LV base-septal (300 µM, LQT1, +55.3±18.5% vs. LMC, +51.5±22.0%, ns) positions.

#### E-4031

**Figure 3:**
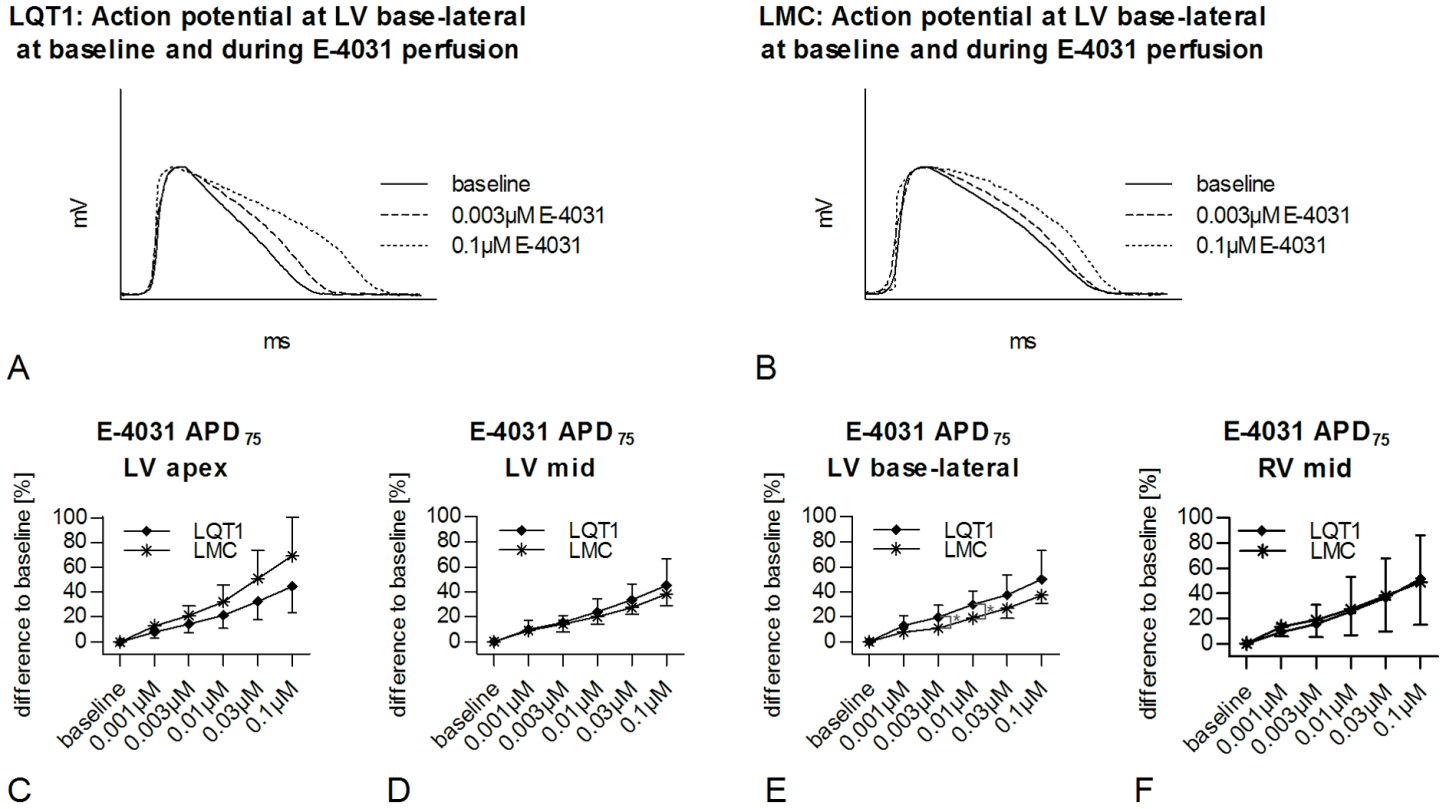
E-4031-induced prolongation of APD in LQT1 and LMC rabbits. Exemplary, representative MAP in (A) LQT1 and (B) LMC rabbits at baseline and at different concentrations of E-4031. Percentaged prolongation of APD related to baseline APD (%APD) in LQT1 (n = 9) and LMC (n = 7) rabbits in (C) LV apex, (D) LV mid, (E) LV base-lateral, and (F) RV mid position during perfusion with increasing concentrations of E-4031. *p<0.05.

Similar to the results in erythromycin-perfused rabbit hearts, a gradually increasing APD_75_ prolongation was seen during perfusion with E-4031 in LQT1 and LMC rabbits in all positions (Figure 3A, 3B). Also, in LV apex, LV mid, and RV mid positions APD_75_ prolongation at low concentrations of E-4031 had a higher level of significance in LQT1 than in LMC rabbits (e.g. LV mid, LQT1, E-4031 vs. baseline [124.5±14.0 ms]; 0.003 µM, 143.4±12.1 ms, p<0.001; 0.1 µM, 180.5±11.0 ms, p<0.001; LMC E-4031 vs. baseline [112.9±4.2 ms]; 0.003 µM, 128.3±9.0 ms, p<0.01; 0.1 µM, 155.3±5.4 ms, p<0.001). Moreover, E-4031 induced an increase of %APD in both, LQT1 and LMC rabbits, but this increase was more pronounced in LQT1 similar to the findings during erythromycin-perfusion: in LV base-lateral position, the increase of %APD was significantly greater in LQT1 than in LMC rabbits (0.01 µM, LQT1, +29.6±10.6% vs. LMC, +19.1±3.8%, p<0.05; Figure 3E). In LV base-septal position, the increase of %APD tended to be more pronounced in LQT1 at the highest concentration of E-4031 (0.1 µM, LQT1, +50.6±23.2% vs. LMC, +30.0±12.5%, p<0.1). In the other positions there was no significant difference between groups (Figure 3D, 3E, 3F).

### Incidence of arrhythmia

During Langendorff-perfusion, only 5 episodes of VT or VF occurred. There was no significant difference in the incidence of arrhythmia between LQT1 and LMC rabbits, neither at baseline nor during perfusion with erythromycin or E-4031.

### Spatial APD dispersion

#### Spatial APD dispersion at baseline

At baseline, intra-ventricular LV APD dispersion (LQT1 vs. LMC, 8.5±3.6 ms vs. 9.1±4.0 ms, ns) and inter-ventricular RV-LV APD dispersion (LQT1 vs. LMC, 19.5±7.9 ms vs. 24.1±4.5 ms, ns) were similar in LQT1 and LMC rabbits.

#### Erythromycin

**Figure 4:**
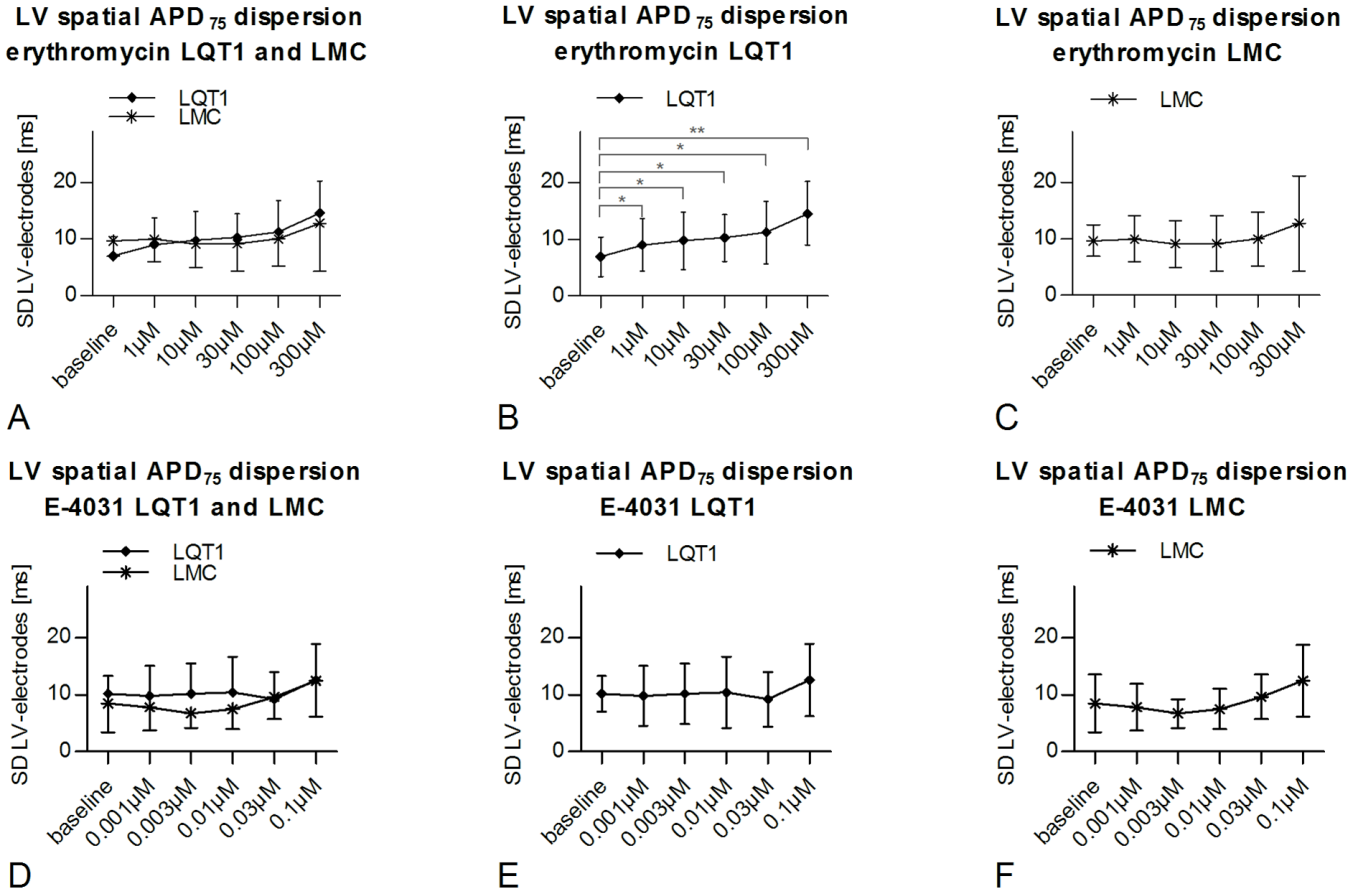
Spatial dispersion of action potential duration in LQT1 and LMC rabbits. Differences of spatial dispersion of action potential duration (APD_75_) between LQT1 (n = 9 for erythromycin, n = 8 for E-4031) and LMC (n = 7 each) rabbits at baseline and during perfusion with erythromycin (A–C) or E-4031 (D–F). t =  trend (p<0.1), *p<0.05, **p<0.01.

In LQT1 rabbits, erythromycin induced a dose-dependent significant increase of individual spatial intra-ventricular LV APD dispersion compared to baseline (paired *t-test*) already at the lowest concentration (erythromycin vs. baseline [7.0±3.5 ms]; 1 µM, 9.0±4.6 ms, p<0.05; 30 µM, 10.3±4.2 ms, p<0.05; 300 µM, 14.6±5.6 ms, p<0.01; Figure 4A, 4B). In LMC, in contrast, no significant change in LV or RV-LV APD dispersion was observed (Figure 4A, 4C). Moreover, erythromycin tended to increase inter-ventricular RV-LV APD dispersion compared to baseline in LQT1 rabbits (300 µM vs. baseline, 23.2±11.5 ms vs. 9.7±5.8 ms, p<0.1). Of note, however, under erythromycin, spatial APD dispersion was not different between LQT1 and LMC rabbits (unpaired *t-test*), due to the fact that baseline APD dispersion was less pronounced (albeit not significantly) in LQT1.

#### E-4031

In contrast to erythromycin, E-4031 did not significantly affect spatial APD dispersion neither in LQT1 nor in LMC (Figure 4D, 4E, 4F).

### Triangulation of monophasic action potential

#### Baseline

**Figure 5:**
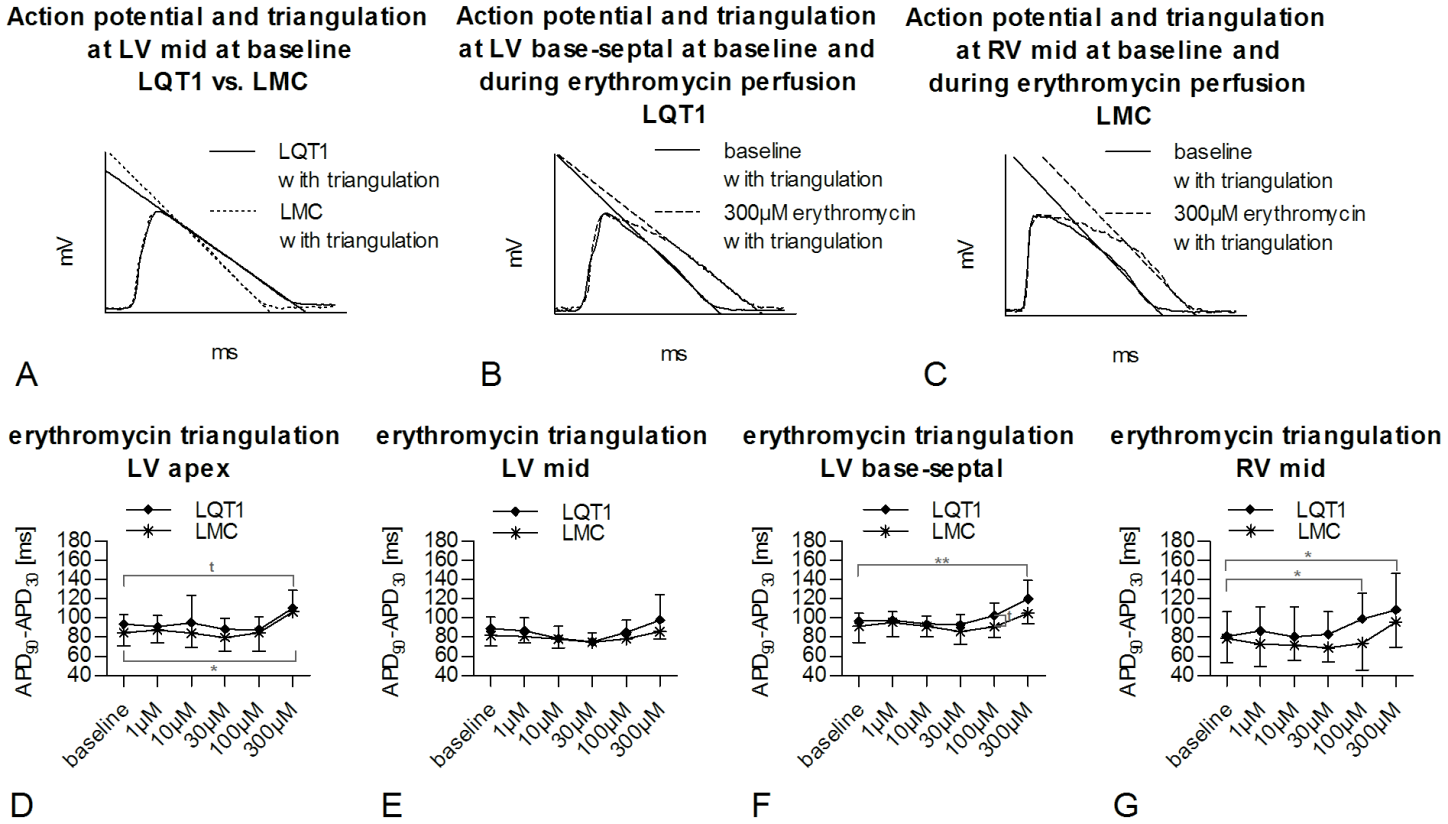
Erythromycin-induced changes in triangulation of monophasic action potential in LQT1 and LMC rabbits. Exemplary, representative MAP triangulation in (A) LQT1 and LMC rabbit at baseline. Exemplary, representative MAP triangulation in (B) LQT1 rabbit and (C) LMC rabbit at baseline and during 300 µM erythromycin. Triangulation of MAP at increasing concentrations of erythromycin in LQT1 (n = 9) and LMC (n = 7) rabbits in (D) LV apex, (E) LV mid, (F) LV base septal, and (G) RV mid position. t =  trend (p<0.1), *p<0.05, **p<0.01.

**Figure 6:**
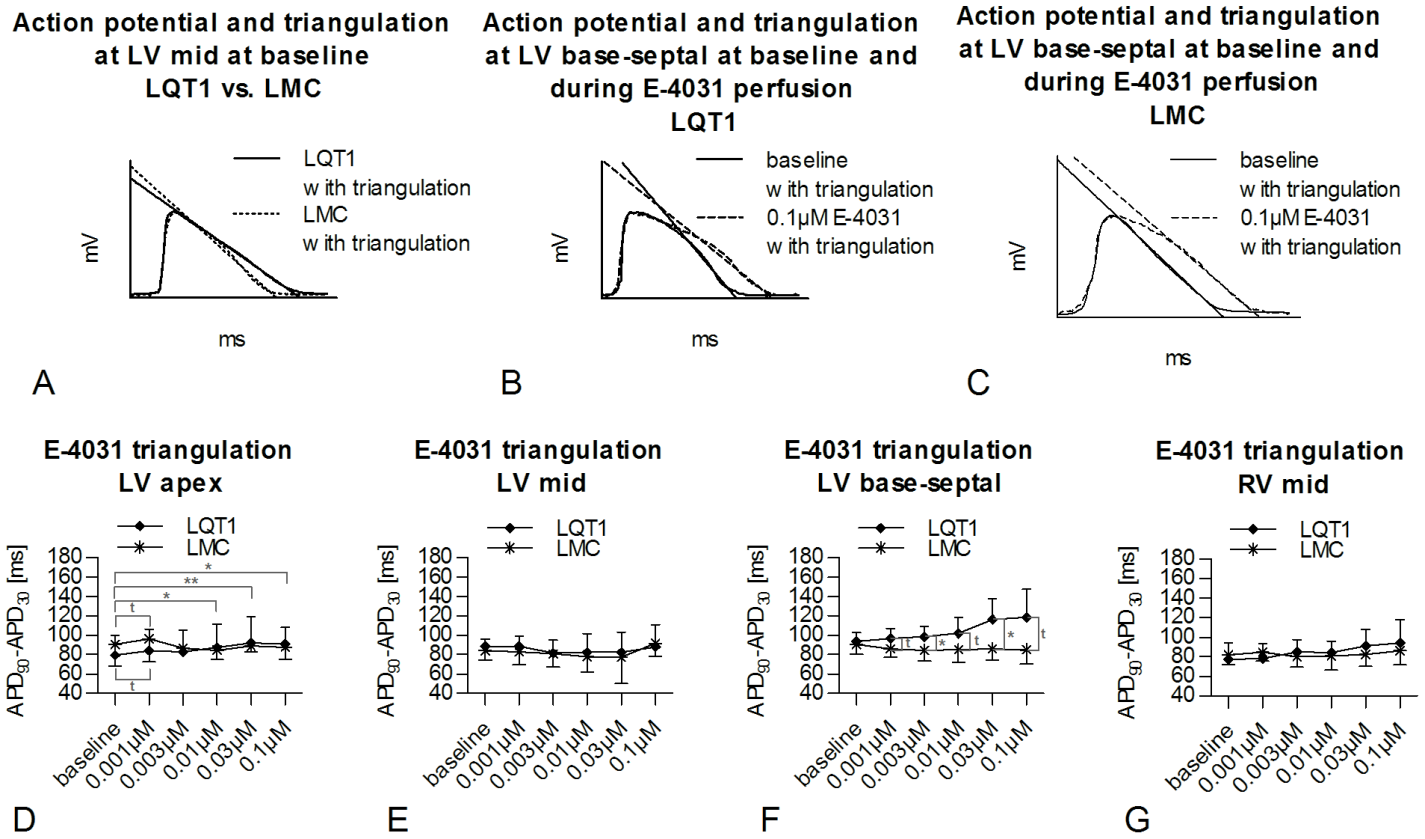
E-4031-induced triangulation of monophasic action potential in LQT1 and LMC rabbits. Exemplary, representative MAP triangulation in (A) LQT1 and LMC rabbit at baseline. Exemplary, representative MAP triangulation in (B) LQT1 rabbit and (C) LMC rabbit at baseline and during 0.1 µM E-4031. Triangulation of MAP at increasing concentrations of E-4031 in LQT1 (n = 9) and LMC (n = 6) rabbits in (D) LV apex, (E) LV mid, (F) LV base-septal, and (G) RV mid positions. t =  trend (p<0.1), *p<0.05, **p<0.01.

Triangulation of the monophasic action potential (MAP) was calculated as a representative parameter for the slowing of phase-3-repolarization (APD_90_ minus APD_30_). At baseline, no significant differences were found in triangulation. A non-significant trend towards a greater triangulation in LQT1 compared to LMC rabbits was only seen in LV mid position (LQT1, 86.4±11.1 ms vs. LMC, 80.3±10.6 ms, p<0.1; Figure 5A, 6A).

#### Erythromycin

During perfusion with erythromycin, an increased triangulation was seen in the LV apex at the highest concentration in both, LQT1 and LMC rabbits (Figure 5D). Additionally, only in LQT1 hearts, a significant erythromycin-induced increase of triangulation was seen in LV base-septal (Figure 5B, 5F) and RV mid (Figure 5C, 5G) positions. In LV mid (Figure 5E) and LV base-lateral positions (300 µM vs. baseline; LQT1, 113.7±32.0 ms vs. 85.6±12.9 ms, ns; LMC, 96.2±20.2 ms vs. 95.1±11.9 ms, ns), however, erythromycin did not increase APD triangulation.

#### E-4031

In LV apex, a significant E-4031-induced increase of triangulation was only seen in LQT1 rabbits (0.1 µM vs. baseline, 91.0±17.2 ms vs. 79.5±20.4 ms, p<0.05; Figure 6D). In LV base-septal position, E-4031-induced triangulation was already greater in LQT1 than in LMC rabbits at a concentration of 0.003 µM (LQT1, 98.5±10.5 ms vs. LMC, 84.5±11.6 ms, p<0.05; Figure 6B, C, F). No significant E-4031-induced alteration of triangulation was observed in the other positions (Figure 6E, G).

## Discussion

Early detection of potentially pro-arrhythmic drugs is a still unmet medical need. In this *proof of concept* study, we have therefore investigated whether transgenic LQT1 rabbits with a reduced repolarization reserve may constitute a sensitive, novel tool for the detection of HERG/*I_Kr_*-blocking properties of drugs, analyzing the impact of the antibiotic drug erythromycin and the class III anti-arrhythmic drug E-4031 on established markers of pro-arrhythmia, such as prolongation of APD, spatial APD dispersion and triangulation.

### APD

Since drugs with HERG/*I_Kr_*-blocking properties typically prolong the APD [19], animal models that sensitively detect HERG/*I_Kr_*-blocking properties of drugs should demonstrate either a particularly pronounced APD prolongation or an APD prolongation already at low drug-concentrations.

Transgenic LQT1 rabbits lack repolarizing *I_Ks_* currents [25] and their repolarization thus mainly depends on *I_Kr_*, conducted by the HERG channel. LQT1 rabbits mimic the human LQT1 phenotype and thus have longer APD and longer QT intervals compared to wild type LMC rabbits already at baseline as demonstrated in our study and previously [25]. Hence, these LQT1 rabbits with a reduced repolarization reserve should sensitively detect APD prolonging properties of HERG/*I_Kr_*-blocking drugs. Both HERG/*I_Kr_*-blocking drugs, erythromycin and E-4031, significantly prolonged APD in both genotypes in all regions of the left and right ventricle, similarly as observed by other groups in wild type rabbit models [20], [30]. The percentaged erythromycin- and E-4031-induced APD prolongation related to baseline APD (%APD), however, was more pronounced in transgenic LQT1 rabbits. Moreover, the erythromycin-induced APD prolongation was spatially heterogeneous in LQT1 rabbits, with a particularly marked APD prolongation in LV base-lateral (300 µM) and RV mid positions (100 µM) at higher concentrations. The more potent HERG/*I_Kr_*-blocker E-4031 exerted a greater APD prolongation in LQT1 than in LMC rabbits at low drug concentrations (0.003 µM and 0.01 µM, LV base-lateral), while a similar APD prolongation was noted in both groups at high concentration of E-4031 (0.03 µM and 0.1 µM).

The maximal concentrations used in our study were 300 µM for erythromycin and 0.1 µM for E-4031. In stably transfected human kidney embryonic (HEK) cells, the IC50 for erythromycin-induced HERG/*I_Kr_*-block was 38.9 µM [31] and 0.008 µM for E-4031 [32]. Thus, likely, E-4031, which is used in a concentration of up to 13x IC50 in the perfusion solution, reaches a sufficient concentration in the cardiac tissue to elicit a complete HERG/*I_Kr_*-block, while erythromycin, used in a concentration of up to 8x IC50, may not necessarily exert a complete block at highest concentration explaining why genotype-specific differences in the susceptibility – with a higher susceptibility in LQT1 rabbits – were obvious only at high concentrations of erythromycin. In E-4031 treated LQT1 hearts, in contrast, a greater E-4031-induced APD prolongation than in LMC rabbits was seen only at low concentrations (0.003 µM and 0.01 µM) in the base-lateral segment, due to a higher susceptibility to E-4031's HERG/*I_Kr_*-block in LQT1 rabbits, while higher concentrations of E-4031 resulted in a complete HERG/*I_Kr_*-block in both, LQT1 and LMC rabbits, thus abolishing genotype differences.

Since drug-induced APD prolongation alone was found to be an insufficient parameter for the detection of drug-induced pro-arrhythmia [19], [23], we have additionally analyzed spatial APD dispersion and triangulation of MAP.

### Spatial APD dispersion

In contrast to the SCREENIT system [19], [33] that applies an endocardial and an epicardial electrode to assess spatial APD dispersion, we derived APD from four LV and one RV electrodes enabling the calculation of intra-ventricular (LV) and inter-ventricular (RV-LV) spatial APD dispersion. Using this method, we demonstrated that at baseline, spatial APD dispersion did not differ significantly between LQT1 and LMC rabbits as also shown *in vivo* (ventricular refractory periods) and *ex vivo* in previous studies in LQT1 rabbits [16], [25], [28] – albeit there was a non-significant trend towards an even less pronounced APD dispersion in LQT1. The lack of differences in APD dispersion at baseline matches data of electrophysiological studies in LQT1 patients, who similarly did not yield greater APD dispersion compared to a healthy control group [34].

In our study, erythromycin dose-dependently increased individual spatial LV APD dispersion as compared to baseline only in LQT1 – but not in LMC rabbits. During erythromycin perfusion, however, overall APD dispersion did not differ between LQT1 and LMC rabbits - due to the slight but non-significant differences in baseline APD dispersion. Since a drug-induced increased spatial APD dispersion compared to baseline may indicate an increased pro-arrhythmic risk of erythromycin [22] as identified in Langendorff-perfused rabbit hearts [20], [33] and in patients treated with antibiotics [35], the occurrence of an erythromycin-induced increased intra- and interventricular APD dispersion only in LQT1 rabbits suggests that LQT1 models may be more sensitive to detect pro-arrhythmic properties of HERG/*I_Kr_*-blockers. Cheng et al. demonstrated an apicobasal dispersion of *I_Kr_* and *I_Ks_* in rabbit cardiomyocytes with a higher current density of *I_Kr_* in the LV apex and a higher current density of *I_Ks_* in the LV base [36]. In the LQT1 rabbit, the absence of the usually predominant *I_Ks_* in the base of the heart could thus explain the sensitivity of a weaker *I_Kr_* current to HERG/*I_Kr_*-block in basal regions. Hence, an erythromycin-induced prolongation of the APD in LV base-lateral position might predominantly lead to the increased LV APD dispersion. Moreover a trend towards a greater RV-LV APD dispersion induced by erythromycin was noted in the LQT1 group. E-4031, however, to our surprise, did not increase spatial APD dispersion in any group. These differences may stem from the fact that E-4031 is a “pure” HERG/*I_Kr_*-blocking agent while erythromycin can also block some other ion channels [37] – albeit to a much lesser degree – which may contribute to the spatial differences in APD prolongation.

### Triangulation

Triangulation of MAP describes the phase-3-repolarization conducted by repolarizing *I_Kr_* and *I_Ks_* currents. An increased drug-induced triangulation indicates that the drug exerts pro-arrhythmic effects, while its absence argues against pro-arrhythmia [19]. An erythromycin- and E-4031 induced increased triangulation, which was associated with pro-arrhythmia, has been described previously [20], [33]. In our study, both, erythromycin and E-4031 increased triangulation only in LQT1 rabbits, presumably due to the reduced repolarization reserve of transgenic LQT1 rabbits. The drugs' effects on triangulation, however, were not uniform throughout the ventricle. Erythromycin enhanced triangulation in LQT1 in LV base-septal and RV mid. Absolute triangulation during erythromycin perfusion, however, did not differ between LQT1 and LMC rabbits. E-4031, in contrast, increased triangulation in the LV apex of LQT1 rabbit hearts and also demonstrated a significantly greater triangulation in the LV base-septal segment in LQT1 hearts compared to LMC rabbits.

### Arrhythmia

Ventricular arrhythmia due to drug-induced HERG/*I_Kr_*-block is a rare but serious side effect of various drugs [3], [4]. In our study, erythromycin and E-4031 induced increased APD dispersion and triangulation, both parameters of pro-arrhythmia. Drug-induced arrhythmia, however, did occur only very rarely. The lack of significant HERG/*I_Kr_*-blocker induced arrhythmia does not surprise, since this study (1.) was designed for the analysis of the MAP, lacking specific ventricular stimulation protocols, (2.) used only small group sizes and (3.) consisted only of a short observation time during the experiments, while drug-induced arrhythmia occur rarely in general. A suitable detection of drug-induced arrhythmia and of a causative context between drugs and cardiac events would require long term *in vivo* telemetric ECG monitoring with continuous drug application.

In non-anaesthetized, free-moving LQT1 rabbits, no increased risk for spontaneous ventricular arrhythmia and sudden cardiac death was observed using telemetric ECG monitoring [25]. In contrast, after AV node ablation and subsequent low frequency pacemaker stimulation (90/min) LQT1 rabbits exhibited a mortality of 50% within 7 days due to ventricular arrhythmia [38], suggesting an anti-arrhythmic effect of fast heart rates, naturally occurring in rabbits, that may protect LQT1 rabbits from drug-induced ventricular arrhythmia *in vivo* and *ex vivo*, if no further pro-arrhythmic factors concur.

## Limitations

*Ex vivo* whole heart animal model are useful and powerful tools for the preclinical safety assessment of drugs [33]. Thus far, however, no single model has demonstrated enough power to sensitively detect pro-arrhythmic effects of HERG/*I_Kr_*-blocking drugs. In this *proof of concept* study, pro-arrhythmic parameters were more pronouncedly changed in LQT1 rabbits – but the study was not powered to detect potential differences in arrhythmic events between LQT1 and LMC rabbits. This stems from the facts that (1.) drug-induced arrhythmia occurs rarely in general and that (2.) other manipulations like induced bradycardia would be required to sufficiently increase the incidence for arrhythmias in the LQT1 rabbits *in vivo* [38] to be able to detect potential differences in drug-induced arrhythmia in relatively small groups of animals. Therefore, additional (long-term) studies in larger groups with further modifications that increase the incidence of drug-induced arrhythmias *in vivo* and on the *ex vivo* whole heart level are clearly warranted to identify the ideal tool to detect pro-arrhythmia reliably. Moreover, to validate the transgenic LQT1 rabbit model as sensitive tool to detect APD prolonging and pro-arrhythmic drug effects, future studies systematically testing several drugs with different degrees of APD-prolonging and pro-arrhythmic properties are of course warranted – ideally, including drugs that exert very little APD prolonging effects in healthy subjects unless concomitant factors impair their repolarization reserve. In a previous study, we could already demonstrated that transgenic LQT1 rabbits may reveal QT-prolonging effects of drugs that do not significantly affect APD/QT in wild type rabbits [27], suggesting that LQT1 rabbits may be particularly sensitive also for drugs that do not prolong cardiac repolarization in “healthy subjects”.

## Conclusions and Clinical Implications

Markers for pro-arrhythmia such as APD prolongation and triangulation were more pronouncedly induced by HERG/*I_Kr_*-blocking drugs erythromycin and E-4031 in LQT1 than in LMC rabbits and the pro-arrhythmic marker increased spatial APD dispersion was induced by erythromycin only in LQT1 rabbits. LQT1 rabbits are thus particularly sensitive to detect HERG/*I_Kr_*-blocking drugs that have previously been classified as pro-arrhythmic. According to the results of the present *proof of concept* study LQT1 rabbits may thus be a sensitive tool for testing drug-induced pro-arrhythmic properties of novel HERG/*I_Kr_*-blocking drugs in the future. Thereby, they may be helpful (1.) to identify drugs with a high arrhythmic risk but also (2.) to identify “safe” drugs. In case a newly tested drug does not prolong APD, induces no indirect pro-arrhythmic markers (APD dispersion, triangulation), and does not increase the incidence of arrhythmia in the more sensitive LQT1 rabbits then the drug may presumably be safe.
